# *Bacillus cabrialesii* BH5 Protects Tomato Plants Against *Botrytis cinerea* by Production of Specific Antifungal Compounds

**DOI:** 10.3389/fmicb.2021.707609

**Published:** 2021-08-06

**Authors:** Lu Zhou, Chunxu Song, Claudia Y. Muñoz, Oscar P. Kuipers

**Affiliations:** ^1^Department of Molecular Genetics, University of Groningen, Groningen, Netherlands; ^2^Key Laboratory of Plant-Soil Interactions, Ministry of Education, College of Resources and Environmental Sciences, National Academy of Agriculture Green Development, China Agricultural University, Beijing, China

**Keywords:** *Bacillus*, tomato, phytopathogens, antifungal compounds, induced systemic resistance

## Abstract

The gray mold caused by the phytopathogen *Botrytis cinerea* presents a threat to global food security. For the biological regulation of several plant diseases, *Bacillus* species have been extensively studied. In this work, we explore the ability of a bacterial strain, *Bacillus cabrialesii* BH5, that was isolated from tomato rhizosphere soil, to control the fungal pathogen *B. cinerea*. Strain *B. cabrialesii* BH5 showed a strong antifungal activity against *B. cinerea*. A compound was isolated and identified as a cyclic lipopeptide of the fengycin family by high-performance liquid chromatography and tandem mass spectrometry (ESI-MS/MS) that we named fengycin H. The fengycin H-treated hyphae of *B. cinerea* displayed stronger red fluorescence than the control, which is clearly indicating that fengycin H triggered the hyphal cell membrane defects. Moreover, root inoculation of tomato seedlings with BH5 effectively promoted the growth of tomato plants. Transcription analysis revealed that both BH5 and fengycin H stimulate induced systemic resistance of tomato plants *via* the jasmonic acid signaling pathway and provide a strong biocontrol effect *in vivo*. Therefore, the strain BH5 and fengycin H are very promising candidates for biological control of *B. cinerea* and the associated gray mold.

## Introduction

Tomato (*Solanum lycopersium*), ranking up to the second important vegetable, is a widely popular grown vegetable throughout the world (Yu et al., 2018). However, a number of diseases impact on the growth and yield of tomato. Gray mold, caused by airborne necrotrophic fungi *Botrytis cinerea*, is one of the most devastating diseases affecting tomato (Asselbergh et al., 2007; Toral et al., 2018). *B. cinerea* is able to attack more than 200 species causing lesions, and sporulating abundantly on aerial parts of plants (Boddy, 2016). Currently, repeated intensive fungicide sprays are necessary to control this pathogen especially in a mild and humid climate (Rupp et al., 2017), due to the lack of cultivars with resistance. Therefore, alternative approaches, less relying on chemicals and more environmentally friendly, are urgently needed for controlling gray mold (Soylu et al., 2010).

Biological control by beneficial microorganisms holds great promise for sustainable agriculture development (Myo et al., 2019). To date, more and more reports show that applying plant growth-promoting rhizobacteria (PGPR) into agriculture as biological control agents is a successful strategy for plant disease control (Ongena and Jacques, 2008). PGPR can influence plant growth by either facilitating resource acquisition or modulating plant hormone levels. Moreover, PGPR can decrease the various pathogenic effects, either by evoking induced systemic resistance (ISR) of plants or by producing antimicrobial compounds (Perez-Garcia et al., 2011; Berendsen et al., 2012; Olanrewaju et al., 2017). Various species from the genus *Bacillus* have been considered biologically safe, and their sporulation capacity makes it possible for them to live in diverse habitats and to be formulated as commercial products (Roh et al., 2007; Ongena and Jacques, 2008; Arguelles-Arias et al., 2009; Perez-Garcia et al., 2011; Toral et al., 2018). *Bacillus* strains can secrete various types of antimicrobial compounds including non-ribosomally synthesized peptides (NRPs), polyketides (PKs), terpenes, and ribosomally synthesized and post-translationally modified peptides which have shown distinct capacities to inhibit plant pathogens (Crits-Christoph et al., 2018). So far, the bioactive lipopeptides produced by *Bacillus subtilis* are mainly classified into three families: surfactin (Arima, 1968; Koumoutsi et al., 2004; Jiang et al., 2016), iturin (Besson et al., 1990; Tsuge et al., 2001), and fengycin (Vanittanakom et al., 1986; Wu et al., 2007; Zhang and Sun, 2018). All of them are cyclic lipopeptides (CLPs), which exhibit antimicrobial and antifungal activity against a broad range of phytopathogens. These CLPs, consisting of a fatty acid chain bound to a cyclic peptide ring, are produced by non-ribosomally synthesized peptide synthetases. Their broad antimicrobial and antifungal spectra offer great potential for plant disease control (Chowdhury et al., 2015). In addition, volatile organic compounds (VOCs) produced by *Bacillus* strains have also been proved to control plant pathogens, *via* inhibiting bacterial and fungal growth, promoting or inhibiting plant growth, triggering plant resistance, and attracting other micro- and macro- organisms (Lim et al., 2017; Tahir et al., 2017a,b; Tyc et al., 2017; Avalos et al., 2018; Xie et al., 2018).

Plants also have their own mechanisms to deal with pathogens and to coordinate the appropriate defense responses. A prominent defense mechanism is dependent on the jasmonic acid (JA) and ethylene (ET) signaling pathway or the salicylic acid (SA) signaling pathway in plants (Pieterse et al., 2009). ISR is an important regulatory strategy of plants, which could activate appropriate cellular defense responses before or upon pathogen attack (Akram et al., 2016). Different PGPRs have been shown to trigger ISR usually relying on the JA and ET signaling pathway (Pieterse et al., 2014). The JA and ET pathways associate with the responses against necrotrophic pathogens and chewing insects, while SA pathway associates with mainly biotrophic pathogens (Martinez-Hidalgo et al., 2015). In general, ISR could result in direct activation of the defense process including the increase of defense-related compound accumulation, and the priming of plant defensive capacity (Van Hulten et al., 2006; Zamioudis and Pieterse, 2012).

*Bacillus cabrialesii* BH5, isolated from tomato rhizosphere soil, showed good antimicrobial activity against *Erwinia carotovora*, *Pseudomonas syringae*, *Rhizoctonia solani*, *B. cinerea*, *Verticillium dahliae*, and *Phytophthora infestans* among 181 other isolates in our previous study (Zhou et al., 2021). Strain BH5 showed antagonistic activity not only against tomato bacterial pathogens but also against tomato fungal pathogens. Therefore, we found it interesting to investigate the interaction mechanism between BH5 and pathogens of tomato plants. In this study, we demonstrate that *B. cabrialesii* BH5 isolated from the rhizosphere soil of a healthy tomato plant, shows potent biocontrol activity against fungal pathogens *via* producing an antifungal compound, that is a variant of fengycin that we named fengycin H, with a different lipid moiety attached. We also found that *B. cabrialesii* BH5 is able to promote tomato plant resistance to *B. cinerea* by activating the jasmonic acid signaling pathway. These findings demonstrate the feasibility of *B. cabrialesii* BH5 to be applied in practice for tomato disease control.

## Materials and Methods

### Strains and Culture Conditions

Strain *B. cabrialesii* BH5 was isolated from the rhizosphere soil of the healthy tomato cultivar Boludo during the Spring (February 2017), grown at Almería in Spain (Zhou et al., 2019, 2021). BH5 was grown on Luria-Bertani (LB) agar plates or LB liquid media at 28°C, 220 rpm. All phytopathogens (*B. cinerea* B05.10, *Fusarium culmorum* PV*, Pythium ultimum* P17, and *R. solani* AG2-2 IIIB) were cultured on Potato Dextrose Agar (PDA) plates or Potato Dextrose Broth (PDB) media at room temperature. Plant pathogens *B. cinerea*, *F. culmorum*, *P. ultimum*, and *R. solani* used in our study were kindly provided by Prof. Jos Raaijmakers of Netherlands Institute of Ecology and Asst. Prof. Jan van Kan of Wageningen University & Research as gifts.

### Antifungal Activity Test

The plant pathogens (*B. cinerea*, *F. culmorum*, *P. ultimum*, and *R. solani*) were grown on PDA plates for 3 days at room temperature. Then, a mycelial plug of 5 mm diameter of each pathogens was placed in the center of a new PDA plate. 5 μl of BH5 culture (OD_600_ = 1.0) was spotted 2 cm away from the plug. LB media only was inoculated as control. The plates were sealed with parafilm and incubated at room temperature for 5 days. Hyphal growth was monitored depending on the pathogens’ growth rate. The percentage inhibition of pathogens growth values was determined according to the equation as below:


$$\begin{array}{l} \mathrm{inhibition rate}\;\left(\%\right)\\ =\frac{Diameterofcontrol-Diameteroftest}{Diameterofcontrol}\times 100 \end{array}$$

### Effect of VOCs From BH5 on Plant Pathogens

The effect of BH5 VOCs on the growth of fungal and oomycetal plant pathogens, such as *B. cinerea*, *F. culmorum, P. ultimum*, and *R. solani*, was investigated using bottoms of two 90-mm-diameter Petri dishes as described before (Cordovez et al., 2015). These two bottoms were sealed facing each other by parafilm and incubated at room temperature. One bottom was used to spread and grow BH5 with LB agar media, another bottom was used to grow fungal or oomycetal pathogen with PDA media. In order to prevent colonies of BH5 falling down on the plates with fungus, the plates of pathogens were turned to the upside once the plug of pathogens stuck on the PDA plates after 2 days. The LB medium plate without BH5 was considered as negative control. Fungal and oomycetal growth inhibition was monitored and measured as the diameter growth of fungal and oomycetal hyphae. Percentage inhibition of pathogens growth values was determined according to the equation as described before.

### Identification of Antifungal Compounds

A single colony of BH5 was inoculated in 5 ml LB broth and incubated at 28°C, 220 rpm for overnight. The overnight culture was diluted at 1:100 in 200 ml LB broth and incubated at 28°C, 220 rpm for 24 h. The supernatant was then collected by centrifugation (10,000 × *g*, 10 min) and precipitated with ammonium sulfate to 40% saturation. The precipitates were dissolved in Milli-Q water and desalted by 10 g C18 silica gel (Sigma-Aldrich). The crude extracts were filter sterilized with 0.45 μm Durapore^™^ membrane and then subjected to a reverse high-performance liquid chromatography (HPLC) equipped with Aeris widepore 3.6 μ XB-C18 250 × 4.6 mm column for purification. The mobile phases were HPLC-grade water supplemented with 0.1% trifluoroacetic acid (TFA; solvent A) and acetonitrile supplemented with 0.1% TFA (solvent B). A two-step gradient of solvent B from 5 to 40% in 10 min and 40 to 95% in 30 min at a flow rate of 1 ml/min was applied for compound purification. A UV-detector set at a wavelength of 214 nm was used to monitor the compounds elution. All the peaks were collected for MALDI-TOF analysis and tandem MS (MS/MS).

### Viability and Integrity Changes of *Botrytis cinerea* Hyphal Cells Caused by the Fengycin H Compound Produced by BH5

Cell viability and cellular integrity were measured by propidium iodide (PI) staining, as described previously with slight modification (Ouyang et al., 2018; Zhang and Sun, 2018). To produce *B. cinerea* mycelium cells, the mycelial plug of 5 mm diameter of *B. cinerea* from PDA plates was inoculated in 50 ml PDB media. After incubation at 28°C for 3 days with shaking at 100 rpm, the purified fengycin H was dissolved in methanol and was injected into 50 ml PDB culture at a final concentration of 20 μm. The resulting cultures were incubated for 48 h. The same amount of methanol was used as the control. The hyphal cells were collected and resuspended with 10 mm phosphate-buffered saline (PBS), and then stained with PI at 28°C for 20 min in the dark. The cell viability and integrity changes were observed and recorded by a fluorescence microscope with a filter of 535 nm/615 nm.

### Characterization of PGPR Related Traits of Strain BH5

**Siderophore production** was investigated using the overlay chrome azurol S assay reported before with slight modification (Perez-Miranda et al., 2007). Briefly, iron-deprived LB media was made by adding 5 g chelex 100 resin into 100 ml LB and stirring for 1.5 h. The overlay of CAS was applied over iron-deprived LB agar plates containing cultivated strain BH5. The ability of siderophore production was evaluated by the size of the orange halo surrounding the colony of isolates after 1 h.

**Protease activity** detection was achieved on 3% (W/V) skimmed milk agar plates (Dinesh et al., 2015). 5 μl 1 × 10^8^ cells/ml overnight culture of BH5 was spot-inoculated at the center of skimmed milk agar plates. After 3 days incubation at 28°C, the radius of the halo zone around the strain was considered as an activity of the protease produced.

**Phosphate solubilization** ability assay was performed on Pikovskaya’s media (PVK media; Ali et al., 2020). The PVK media plates were spot-inoculated at the center of 5 μl 1 × 10^8^ cells/ml overnight culture of BH5 and incubated at 28°C for 7–10 days. The zone of Phosphate solubilization surrounding the colonies was measured.

**The Indole-3-acetic acid production** assay was carried out with the protocol reported previously (Bric et al., 1991; Goswami et al., 2013, 2014). The capacity of indole-3-acetic acid (IAA) biosynthesis of isolates was estimated by the Salkowski colorimetric technique with slight modifications. BH5 was grown in LB media supplemented with 5 mm tryptophan for 3 days. Subsequently, supernatants were collected by centrifugation at 10,000 x g for 30 min. 2 ml supernatant was mixed with 10 μl of orthophosphoric acid and 4 ml Salkowski reagent. After 25 min incubation at room temperature, the intensity of pink color was detected at 530 nm spectrophotometrically and the amount of IAA was calculated based on the standard curve.

**Swarming motility** was investigated on LB plates supplemented with 0.7% agar (Gao et al., 2016). 5 μl 1 × 10^8^ cells/ml overnight culture of BH5 was spot-inoculated at the center of 0.7% agar swarming plates and incubated at 28°C overnight and the zone of colonization on 0.7% agar swarming plates was measured.

**The biofilm formation** ability was tested according to a previous protocol (Oliveira et al., 2015; Zhao et al., 2015). The growth of biofilm was quantified by the crystal violet staining method in 96-well polystyrene plates. Briefly, an overnight culture (1:100) of BH5 was inoculated in 100 μl LB media and incubated at 30°C for 24 h. Then, the culture was stained with 40 μl 0.25% crystal viollet for 15 min. After a three times wash with PBS buffer, to each well was added 200 ul 33% acetic acid to dissolve the biofilm for 15 min. The optical density was measured at OD_595_ and the value was used as a proxy for biofilm formation.

### *In vivo* Plant Growth Promotion

Tomato seeds (Moneymaker) were surface disinfected in 70% ethanol for 1 min and 2% NaClO solution for 10 min. Subsequently, tomato seeds were rinsed five times with sterilized deionized water. Seeds were pre-germinated horizontally on 15 cm Petri dishes with Murashige and Skoog (MS) agar in a growth chamber (16 h day time at 24 ± 2°C, 8 h dark time at 21 ± 2°C) for 2 days. Pre-germinated seeds were transferred to new MS agar plates and incubated vertically in a growth chamber for 2 days. BH5 was grown in LB media at 28°C and 220 rpm for overnight. Cells were harvested and then washed with Phosphate buffer solution (PBS) and diluted to a final concentration of 1 × 10^7^ cells/ml with appropriate PBS. Five-day-old root tips of seedlings were inoculated with 5 μl of cell suspension. The effects of bacteria on the tomato plants were evaluated 3 weeks post-inoculation.

### Assessment of BH5 Protective Effect on Tomato Plants Against *B. cinerea*

One-month-old tomato (Moneymaker) plants were used for antifungal bioassays. These plants were grown in plastic pots of 1 L capacity, filled with sterilized commercial substrate and placed in a growth chamber at 24/21°C with a 16/8 h photoperiod and 60% humidity. The inoculation was performed 3 days before pathogen challenge. 5 ml of BH5 suspension (10^9^ cells/ml) in PBS buffer or 5 ml purified pure fengycin H (20 μm) in PBS buffer was carefully brought in the soil near the roots of the tomato plant using a micropipette, while the same amount of PBS buffer was used as control. All the treatments were divided into two groups. One group of treatments was sampled at 0, 24, 48, 72, 96, and 120 h for the measurements of the relative expression of JA signaling pathway-related genes. Another group was subjected to *B. cinerea* challenge. For antifungal assay *in vivo*, after 3 days inoculation of BH5 or the fengycin H, tomato leaves were collected and washed with sterilized Milli-Q water, then subjected to antifungal bioassay. Three leaves of each plant were placed in plastic trays on wet paper and challenged with *B. cinerea* by applying 5 mm diameter plugs of one-week-old PDA culture plates. The leaves were then placed in a growth chamber. The disease symptoms were scored on each leaf after 2 days of infection and thus recorded every 2 days over a 15-day period. The diameter of the necrotic lesions formed by the fungal hyphae in the leaflets was measured. The biocontrol efficacy was calculated by the formula reported before (Soylu et al., 2010). Biocontrol $\mathrm{Efficacy}=\left[\left(\mathrm{Lc}-\mathrm{Lt}\right)/\mathrm{Lc}\right]\times 100$, where Lc indicating lesion diameter in control and Lt indicating lesion diameter in treatment.

### Quantitative Real-Time PCR Analysis

A 100 mg sample of frozen leaves in powder form was used to extract the total RNA with the RNeasy Plant Mini Kit (QIAGEN, Germany). The total RNA was dissolved in 30 μl of RNAse-free water, quantified by microspectrophotometry, and stored at −80°C. In brief, 1 μg total RNA was used for cDNA synthesis by using SuperScript^™^ III Reverse transcriptase (Invitrogen) following the instruction manual. After the cDNA was diluted 10 times, 2 μl diluted cDNA was used for quantitative real-time PCR (qRT-PCR) with the following program: 95°C 3 min, followed by 40 cycles at 95°C for 30 s, 57°C for 30 s, and 72°C for 30 s. The relative gene expression for each sample was normalized and calibrated to the housekeeping gene *SlEF* (encoding for the tomato elongation factor-1α) *Ct* value, and counted using the formula 2^−ΔΔ*Ct*^ (Cheng et al., 2017). All experiments were run in triplicate with different cDNAs synthesized from three biological replicates. Three (*SlLoxD*, *SlPI II*, and *SlJAZ1*) genes related to JA signaling pathway were selected for qRT-PCR analysis. Primers for qRT-PCR are listed in Table 1. The *SlLoxD* gene (encoding for a JA-inducible lipoxygenase) is related to the biosynthesis of JAs, involving in the generation of endogenous jasmonic acid (JA), and regulating the expression of plant defense genes and resistance to high temperature and pathogen attack (Hu et al., 2015; Beris et al., 2018). The *SlPI II* gene (encoding for proteinase inhibitor II), mainly regulated by JA and MeJA, is a marker of JA/ET-dependent pathways (Xin et al., 2014). The role of *SlPI II* gene is making a contribution in plant defense against herbivores and pathogens (Rehman et al., 2018). *SlJAZ1*, which is a negative gene in the JA signaling pathway (Thines et al., 2007; Chico et al., 2008), encoding for key repressors in the JA signaling pathway and acting as a primary role of tomato in cell death associated to pathogen infections (Ishiga et al., 2013; Chini et al., 2017).

**Table 1:** Sequences of specific primers used for qPCR analysis.

Gene name	Accession number	Forward primer (5'-3')	Reverse primer (5'-3')
*SlEF* (housekeeping)	X53043	GGTGCATCAAATTCTGCCTGT	GGCGACTTGGTTGTCACTCT
*SlLoxD*	NC_015440.3	GGCACGGTGTCACTGTTATT	CATAAGGCTGTGTTGCGTCC
*SlPI II*	NM_0012477698	TTCGGGATATGCCCACGTTC	GGTGCAAGCATTTGGCCTTT
*SlJAZ1*	NM_001247954	AGCCAACAAACAGAACCCCA	AATTCCGTCTCGCGATTGGT

## Results

### Antifungal Activity of *B. cabrialesii* BH5

**Figure 1:**
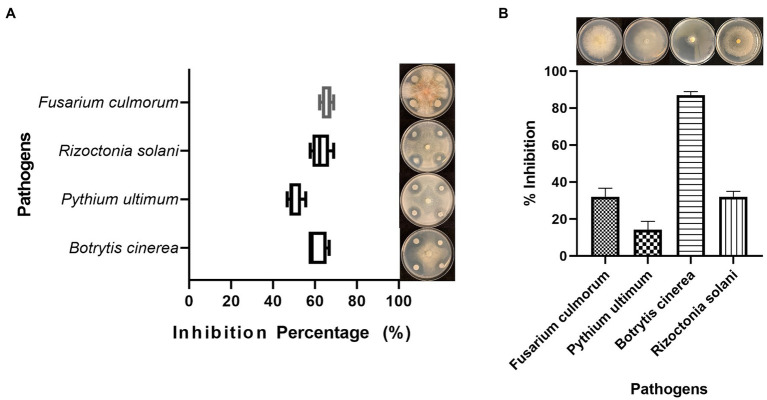
Antifungal activity of *Bacillus cabrialesii* BH5 toward four plant fungal pathogens **(A)** and the antifungal activity of volatile organic compounds produced by BH5 **(B)**.

Antifungal activity was detected by the dual culture method on PDA plates. *B. cabrialesii* BH5 showed a strong ability to inhibit the growth of four test pathogens (Figure 1A). The mycelial inhibition rates were 61, 66, 50, and 63% toward *B. cinerea*, *F. culmorum, P. ultimum*, and *R. solani*, respectively.

### Antifungal Activity of VOCs Produced by *B. cabrialesii* BH5 on Agar Media

To investigate the effect of VOCs produced by BH5 on plant pathogens, we performed the so-called sandwich assay (Cordovez et al., 2015). Once the hyphae reached the edge of the plates in control plates, the hyphal growth of *B. cinerea*, *F. culmorum, P. ultimum*, and *R. solani* was measured and recorded. BH5 showed the inhibition on the hyphal growth of pathogens (Figure 1B). The inhibition rates were 87, 32, 14, and 32% toward *B. cinerea*, *F. culmorum, P. ultimum*, and *R. solani*, respectively.

### Antifungal Activity of the Lipopeptides Produced by *B. cabrialesii* BH5

**Figure 2:**
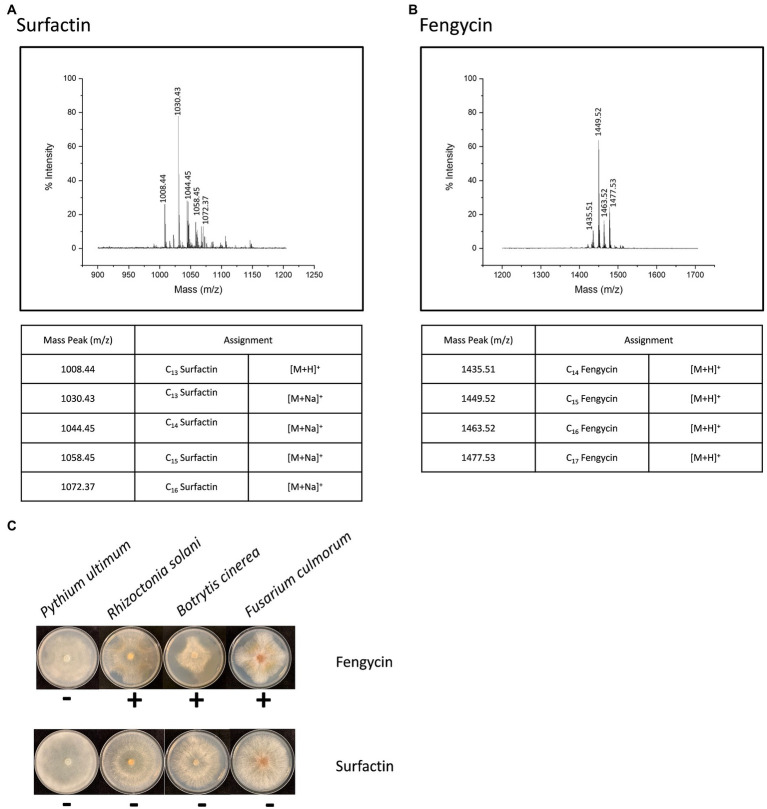
Two families of lipopeptides produced by *B. cabrialesii* BH5. The ensembles found in the mass ranges m/z 1008–1072 **(A)** and m/z 1435–1477 **(B)** were attributed to the well-known surfactins and fengycins. Effect of surfactins and fengycins produced by BH5 on four different plant pathogens **(C)**.

**Figure 3:**
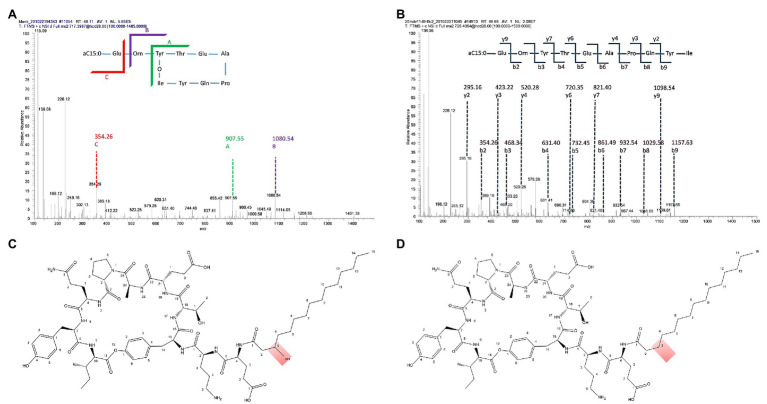
Characterization of fengycin H produced by *B. cabrialesii* BH5. **(A)** Tandem MS analysis of fengycin H. **(B)** Tandem MS analysis of hydrolyzed fengycin H (fengycin H was hydrolyzed with 2 M NaOH). **(C)** The structure of C15 fengycin. **(D)** The structure of fengycin H produced by BH5.

Two product families were detected in the extracts (Figure 2). They were found in the mass ranges m/z 1008–1072 (Figure 2A) and m/z 1435–1477 (Figure 2B) and were attributed to the well-known surfactins and fengycins. These two families of lipopeptides were then subjected to investigate the activity with phytopathogens *in vitro*. Only the fengycins showed bioactivity against the hyphal growth of *B. cinerea*, *F. culmorum*, and *R. solani* (Figure 2C). To further characterize the antifungal compounds, all the peaks were applied to LC-MS/MS analysis. A fengycin-like compound (m/z 1433.8) was found in the extract (Figures 3A,B). This compound showed identical amino acid composition as fengycin A but had a different fatty acid chain. It is different from fengycins discovered until now, because the lipid portion of fengycins consists of a β-hydroxy fatty acid, with its-COOH group amide bonded to α-NH_2_ of Glu1 (Figure 3C). However, there is no β-hydroxyl at the fatty acid chain in the fengycin-like compound produced by BH5 as shown in Figure 3D, which makes the chain more hydrophobic. We denoted this variant as fengycin H.

### Effect of Fengycin H on *B. cinerea* Hyphal Cell Viability and Cellular Integrity

**Figure 4:**
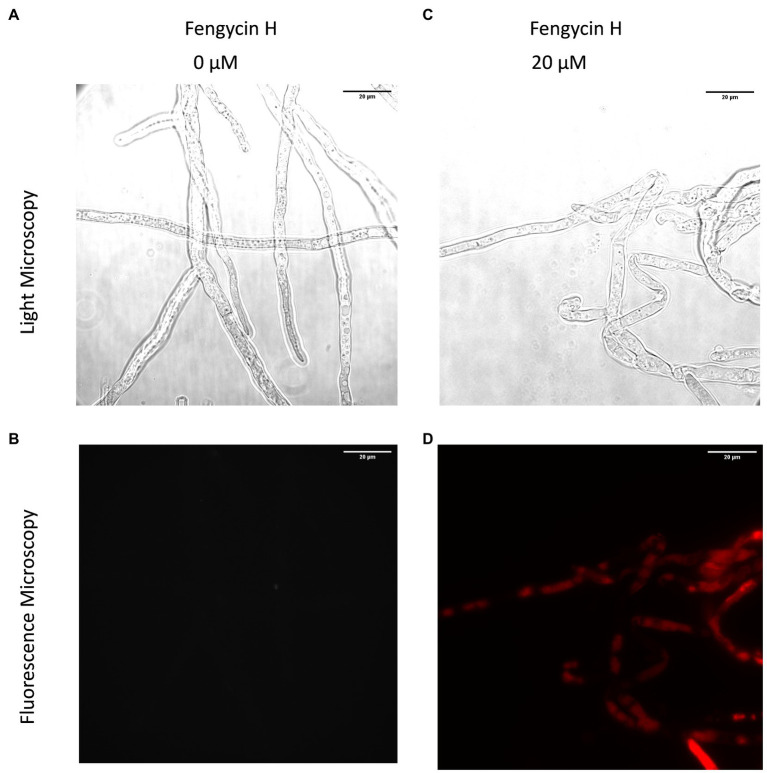
Effect of fengycin H from *B. cabrialesii* BH5 on viability and membrane integrity of *Botrytis cinerea* hyphal cells, observed by light microscopy **(A,C)** and fluorescence microscopy **(B,D)**. Live fungal hyphal cells with intact membranes show no fluorescence under fluorescence microscopy **(B)**; the damaged cellular membranes of fungal hyphae showed red fluorescence **(D)**. Methanol served as the control treatment.

Hyphal cell viability and cellular integrity were investigated by the fluorescent dye propidium iodide (PI) which is excluded from viable cells. Cells with a compromised membrane integrity take up the probe and appear as red labeled upon binding of the dye to DNA as shown in Figure 4. The fengycin H-treated hyphae of *B. cinerea* displayed stronger red fluorescence than the control, which is clearly indicating that fengycin H triggered the hyphal cell membrane defects and cell death.

### Assessment and Characterization of PGPR Traits of *B. cabrialesii* BH5

BH5 was screened for diverse PGPR traits, such as siderophore production, protease activity, phosphate solubilization, IAA production swarming motility, and biofilm formation ability. As shown in Supplementary Table S1 and Supplementary Figure S1, BH5 was positive for siderophore production, protease production, phosphate solubilization, swarming motility, and biofilm formation. In addition, the IAA production by BH5 was measured by Salkowski assay, and IAA was produced at 5.8 ± 0.2 μg/ml.

### Tomato Plant Growth Promotion by *B. cabrialesii* BH5

**Figure 5:**
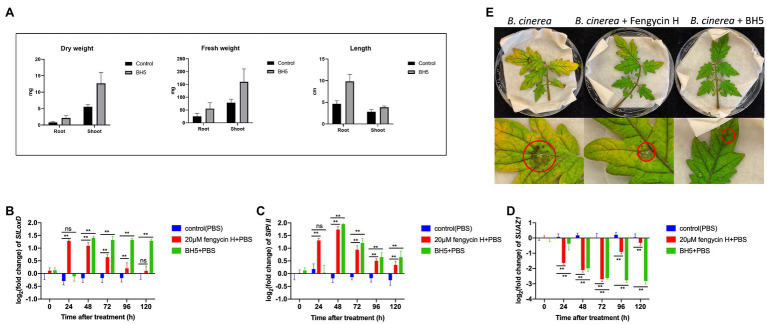
Plant growth promotion activity of *B. cabrialesii* BH5 on tomato seedlings **(A)** and effects of fengycin H and BH5 treatment on JA signaling-related gene expressions: **(B)** *SlLoxD*, **(C)** *SlPI II*, and **(D)** *SlJAZ1*. Significant differences between 20 μm fengycin H or strain BH5 treatment group and control group were compared by Duncan‘s multiple range test. Significant differences at *p* < 0.01 and *p* < 0.05 were marked as (^**^) and single (^*^), respectively. The data (mean ± SD) were calculated using five replicate assays. ns means no significant difference was detected. Test of *B. cinerea* pathogenicity on detached leaves over 5 days **(E)**.

In Figure 5A, the effect of BH5 on the growth of tomato seedlings was monitored by measuring the shoot/root length, shoot/root fresh weight, and dry weight. *B. cabrialesii* BH5 showed a significantly increase of fresh and dry weight both of the shoot and root. The fresh weight of shoot and root significantly increased by about 97 and 30 mg, respectively, as compared to the control. The dry weight of shoot and root significantly increased by about 7.1, 1.3 mg, respectively, as compared to the control. The length of the root significantly increased by about 5.18 cm as compared to the control, while the length of the shoot showed no significantly increase as compared to the control.

### Transcription Analysis of Genes Involved in the JA Signaling Pathway in Tomato Leaves

To investigate ISR stimulated by BH5 or fengycin H in our study (Figures 5B–D), the expressions of three genes related to the JA signaling pathway of tomato at 0, 24, 48, 72, 96, and 120 h after treatment were measured. For the JA signaling pathway-related gene *SlLoxD*, the results (Figure 5B) showed that the expression of *SlLoxD* gene in fengycin H or BH5 treatment was always significantly higher than that in control from 48 to 96 h, and the expression of the *SlLoxD* gene in fengycin H treatment peaked at 24 h and then decreased. Meanwhile, the expression level of *SlLoxD* gene under BH5 treatment started to increase at 48 h, whereas the expression of *SlLoxD* in fengycin H treatment showed no significant difference compared with the control at 120 h. For the JA signaling pathway-related gene *SlPI II* (Figure 5C), we found that the transcript levels of *SlPI II* gene were significantly higher in plants inoculated with fengycin H or BH5 from 48 to 120 h, and both of them peaked at 48 h. Remarkably, the relative expression of *SlPI II* gene of tomato plants inoculated with fengycin H was significantly higher than that inoculated with BH5 at 24 h and significantly lower than that inoculated with BH5 from 48 to 120 h. Additionally, for the JA signaling pathway-related gene *SlJAZ1* (Figure 5D), the variable patterns of *SlJAZ1* expressions in fengycin H and BH5 treatments tomato plants significantly decreased from 24 h.

### Bioassay Against *B. cinerea* on Detached Tomato Leaves

The efficiency of BH5 and fengycin H for controlling *B. cinerea* in tomato plants was assessed on detached leaves. An obvious difference in virulence between the control and treatments was observed (Figure 5E). Results from the bioassay showed that BH5 and fengycin H prevented disease symptoms caused by *B. cinerea* on tomato leaves. After 2 days infection, the leaves turned yellow and exhibited necrotic spots in control. In contrast, leaves treated with BH5 or fengycin H only started to appear small necrotic spots after 6 days infection. The biocontrol efficacies were 71.4% for BH5 treatment and 57.1% for fengycin H treatment. The average lesion diameter was 7.0 ± 0.3 mm in the control, while 2.0 ± 0.3 and 3.0 ± 0.2 mm were measured both on BH5 and fengycin H treatments.

## Discussion

The definition of PGPR is well established and it is therefore worth considering the relationship between PGPRs and biocontrol. By suppressing well-known diseases caused by major pathogens or reducing the deleterious effects of pathogens, PGPR indirectly increases plant growth (Whipps, 2001; Aktan and Soylu, 2020). Moreover, PGPR also increases plant development directly, such as by associative N_2_ fixation (Khan, 2005), solubilizing nutrients (phosphate solubilization; Jeon et al., 2003), producing siderophore (Bhattacharyya and Jha, 2012), releasing phytohormones (IAA production; Marques et al., 2010), and decreasing the toxicity of heavy metals (Bhattacharyya and Jha, 2012). For plant disease control, earlier research has repeatedly documented that natural antagonistic microorganisms can regulate many plant diseases through the production of antipathogenic compounds, competition for space and other necessities, or stimulating the host defense mechanism (Cook and Baker, 1983; Cook et al., 2002).

In this work, we report the biocontrol potential of *B. cabrialesii* BH5 for gray mold in tomato as well as its plant growth-promoting ability. BH5 shows positive traits of PGPR (Supplementary Table S1) and can promote tomato seedlings growth in the climate chamber (Figure 5A). These findings are the first records of *B. cabrialesii* that shows both *in vivo* and *in vitro* antagonistic activity against *B. cinerea*. Besides, we also demonstrate both BH5 and its antifungal compound (fengycin H) can stimulate ISR of tomato plants (Figures 5B–D).

The synthesis of NRPs, such as iturin (Besson et al., 1990), fengycin (Wu et al., 2007), and surfactin (Arima, 1968), is one of the major factors linked to the antifungal activity of members of the genus *Bacillus* (Soylu et al., 2005; Romero et al., 2007; Ongena and Jacques, 2008; Pramudito et al., 2018). Q-TOF MS analysis was performed to detect the lipopeptides released by BH5, showing that strain BH5 produces all lipopeptides except iturin. Some of these macromolecules have been shown to be formed by *B. velezensis* strains (Rabbee et al., 2019). In our research, only fengycins showed good antifungal activity against phytopathogens, including *B. cinerea*, *F. culmorum*, and *R. solani* (Figure 2C). The PI staining assays further indicate that fengycin H discovered from BH5 was responsible for cell wall integrity defects and finally the death of these hyphal cells (Figure 4). Overall, our findings indicate that fengycin H causes significant plasma membrane damage in the fungal pathogen *B. cinerea*, which results in the death of cells. Furthermore, our study of the BH5 and its fengycin H treatments of tomato plants before *B. cinerea* challenge showed that both BH5 and fengycin H could stimulate ISR of tomato plants by JA signaling pathway (Figures 5B–D). JAs are one of the most essential plant pathogen defense hormones, and previous studies have shown that plants can activate the pathway of JA signaling in resistance to necrotrophic pathogens (Trusov et al., 2009; Nie et al., 2017). Mutant plants with JA signaling defects were more susceptible to *B. cinerea* (Rossi et al., 2011). The *SlLoxD* gene (encoding for a JA-inducible lipoxygenase; Beris et al., 2018) is related to the biosynthesis of JAs. The *SlPI II* gene (encoding for proteinase inhibitor II; Xin et al., 2014), mainly regulated by JA and MeJA, is a JA-dependent gene. In our research, upregulated expression of *SlLoxD* and *SlPI II* genes in tomato plants treated with fengycin H and BH5 showed that both fengycin H and BH5 may boost gene expression associated with biosynthesis of JAs to promote disease resistance in tomato plants. Furthermore, decreased transcript levels of *SlJAZ1*, which is a negative gene in the JA signaling pathway (Thines et al., 2007; Chico et al., 2008), in fengycin H and BH5 treated tomato plants compared with the control further confirmed that both fengycin H and BH5 could activate JA signaling pathway to enhance disease resistance to *B. cinerea* in tomato plants. Interestingly, *SlLoxD* and *SlPI II* genes of tomato plants under BH5 treatment always exhibit 24 h later an upregulated or downregulated expression as compared to fengycin H treatment, which is likely to suggest that BH5 will take 2 days after inoculation to successfully colonize the tomato root. This is relatively quicker than other PGPR, with this vigorous capacity for occupation that could effectively allow BH5 colonize the root area against plant exudates and other rhizobacteria. When *B. cinerea* was applied to detached leaves after 3 days inoculation of fengycin H or BH5, disease severity is significantly reduced. This result demonstrates that the durable systemic resistance induced by BH5 or fengycin H could continuously provide a disease control ability for tomato. Altogether, the *in vivo* and *in vitro* investigation of biocontrol ability of fengycin H and strain BH5 suggest an important role of BH5 for use as biocontrol agent against *B. cinerea* as well as a biofertilizer for promoting tomato growth. Various *Bacillus* spp. strains stimulating plant defense reactions have been reported. The existence of molecular determinants secreted by these Gram-positive bacteria responsible for the elicitation of the phenomenon of ISR is, however, still little understood. This study shows that the fengycin H lipopeptide may be involved in this process of elicitation. We demonstrated that the ISR-mediated protective effect on tomato plants can be achieved by pure fengycin H in tomato plants *via* the JA signaling pathway. This finding is consistent with a previous study which reported that pure fengycins and surfactins can provide a significant ISR-mediated protective effect on bean plants (Ongena et al., 2007). By transcription analysis, we provide the evidence that fengycin H from BH5 stimulates the ISR in tomato plants *via* the JA signaling pathway.

## Data Availability Statement

The original contributions presented in the study are included in the article/Supplementary Material, and further inquiries can be directed to the corresponding author.

## Author Contributions

LZ and OK conceived the study and designed the experiments. LZ conducted the experiments and wrote the draft manuscript. LZ, CS, CM, and OK corrected the manuscript. All authors have read and approved the manuscript.

## Conflict of Interest

The authors declare that the research was conducted in the absence of any commercial or financial relationships that could be construed as a potential conflict of interest.

## Publisher’s Note

All claims expressed in this article are solely those of the authors and do not necessarily represent those of their affiliated organizations, or those of the publisher, the editors and the reviewers. Any product that may be evaluated in this article, or claim that may be made by its manufacturer, is not guaranteed or endorsed by the publisher.
