# Supplementary material for: Bacillus cabrialesii BH5 Protects Tomato Plants Against Botrytis cinerea by Production of Specific Antifungal Compounds
Source: Front Microbiol. 2021 Aug 6;12:707609. doi: 10.3389/fmicb.2021.707609 (PMC8441496; doi:10.3389/fmicb.2021.707609)
Supplement: Supplementary file 1 [file Table_1.DOCX]

**Supplementary**

Table S1. Summary of PGPR traits of strain *B. cabrialesii* BH5

| Strain | PGPR traits | | | | | |
| --- | --- | --- | --- | --- | --- | --- |
|  | Siderophore  production | Protease production | Phosphate solubilization | IAA  production | Swarming | Biofilm formation |
| *B. cabrialesii* BH5 | + | + | + | 5.75 ± 0.19 μg/mL | + | + |
