# Supplementary material for: Bacillus cabrialesii BH5 Protects Tomato Plants Against Botrytis cinerea by Production of Specific Antifungal Compounds
Source: Front Microbiol. 2021 Aug 6;12:707609. doi: 10.3389/fmicb.2021.707609 (PMC8441496; doi:10.3389/fmicb.2021.707609)
Supplement: Supplementary file 2 [file Image_1.PDF]

Supplementary

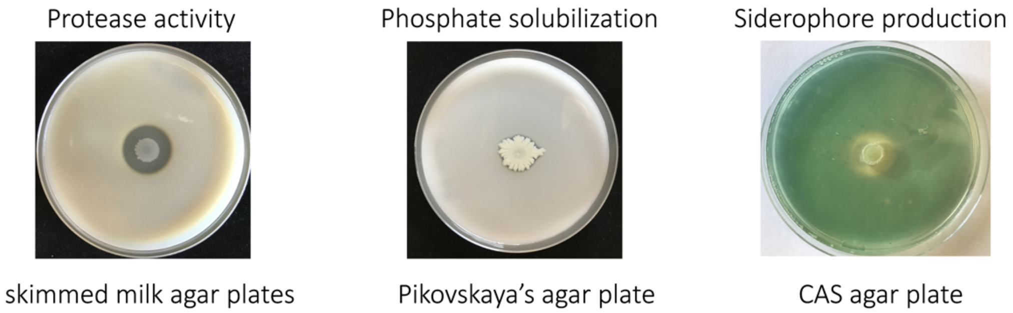

Figure S1. Pictures of *B. cabrialesii* BH5 with clear halo on SMA, PVK or CAS agar plates.
